# Bacterial Succession through the Artisanal Process and Seasonal Effects Defining Bacterial Communities of Raw-Milk Adobera Cheese Revealed by High Throughput DNA Sequencing

**DOI:** 10.3390/microorganisms9010024

**Published:** 2020-12-23

**Authors:** José M. Ruvalcaba-Gómez, Raúl J. Delgado-Macuil, Lily X. Zelaya-Molina, Otoniel Maya-Lucas, Edmundo Ruesga-Gutiérrez, Luis M. Anaya-Esparza, Zuamí Villagrán-de la Mora, David A. López-de la Mora, Ramón I. Arteaga-Garibay

**Affiliations:** 1 Campo Experimental Centro Altos de Jalisco, Instituto Nacional de Investigaciones Forestales, Agrícolas y Pecuarias, Av. Biodiversidad # 2470, Tepatitlán de Morelos, Jalisco 47600, Mexico; ruvalcaba.josemartin@inifap.gob.mx; 2 Centro de Investigación en Biotecnología Aplicada, Instituto Politécnico Nacional, Carretera Estatal Santa Inés Tecuexcomac–Tepetitla, km. 1.5, Tepetitla de Lardizábal, Tlaxcala 90700, Mexico; rdelgadom@ipn.mx; 3 Centro Nacional de Recursos Genéticos, Instituto Nacional de Investigaciones Forestales, Agrícolas y Pecuarias, Blvd. de la Biodiversidad #400, Tepatitlán de Morelos, Jalisco 47600, Mexico; zelaya.lily@inifap.gob.mx; 4 ATGenomics, Ciudad de México 06300, Mexico; otto94@gmail.com; 5 Centro Universitario de los Altos, Universidad de Guadalajara, Av. Rafael Casillas Aceves 1200, Tepatitlán de Morelos, Jalisco 47620, Mexico; edmruesga@gmail.com (E.R.-G.); luis.aesparza@academicos.udg.mx (L.M.A.-E.); blanca.villagran@academicos.udg.mx (Z.V.-d.l.M.); 6 Centro Universitario de Tonalá, Universidad de Guadalajara, Av. Periférico Norte No. 555, Tonalá, Jalisco 48525, Mexico; david.lopezdelamora@cutonala.udg.mx

**Keywords:** genuine Mexican cheeses, artisanal dairy, next-generation sequencing, *Streptococcus*

## Abstract

The bacterial community of the artisanal Adobera cheese from Los Altos de Jalisco was described through high-throughput sequencing of 16S rRNA gene libraries. Samples were collected in two different seasons (dry and rainy) during four key steps of the manufacturing process (raw milk, fresh curd, matured curd, and cheese). Bacterial diversity was higher in early steps in comparison with the final elaboration stages. Firmicutes and Proteobacteria were the most abundant phyla, strongly represented by the *Streptococcaceae*, *Enterobacteriaceae* and *Lactobacillaceae* families, and core bacteria genera such as *Streptococcus* spp., *Lactococcus* spp., and *Lactobacillus* spp. Undesirable bacteria, including *Pseudomonas* spp. and *Acinetobacter* spp., were also detected in raw milk but almost undetectable at the end of the cheese manufacturing process, and seemed to be displaced by lactic-acid bacteria-related genera. Seasonal effects were observed on the community structure but did not define the core microbiota composition. Predictive metabolism was related to membrane transport, and amino-acid, lipid, and carbohydrate metabolism pathways. Our results contribute to deduce the role of bacteria involved in Adobera cheese manufacturing in terms of the metabolism involved, cheese microbial safety, and how undesirable bacterial populations could be regulated by process standardization as a potential tool to improve safety.

## 1. Introduction

Fermentation is traditionally used to improve the organoleptic, nutritional, and beneficial properties of a product through the action of microorganisms. Therefore, fermented foods have been defined as processed foods or beverages produced through controlled microbial growth and enzymatic conversions of their components [1,2]. Fermentation is used to produce several dairy products, cheese being the most popular, because the production of both fresh (soft) and ripened (hard) cheeses is based in milk fermentation for a certain time. Cheeses can be made by artisanal (fermentation associated with the native milk microbiota) or standardized processes (inoculation of defined bacterial cultures). The latter are distinguished by the consistent quality of the release products. In contrast, the quality of artisanal cheeses is heterogeneous (seasonal and batch effects); their production is limited to certain niches; and they are usually made by processes that do not involve sophisticated technology and even are produced within a farm, a town or a specific locality, besides being a unique expression of the interaction between human resources, culture, and nature [3].

In particular, artisanal cheeses (made from raw milk) are usually composed of complex microbial communities associated with the available nutrients, moisture, pH, and lack of pasteurization or some treatment aimed to reduce the microbial content of raw milk [4,5]. Some studies based on culture-dependent microbiology confirm that raw-milk microbiota are usually represented by the following bacteria genera: *Lactobacillus*, *Streptococcus*, *Enterococcus*, *Lactococcus*, *Leuconostoc*, *Weisella*, and *Pediococcus,* in addition to some fungi and yeasts [5,6]. On the other hand, culture-independent techniques have been able to describe not only the cultivable fraction of matrices such as milk and cheese, but to determine the composition of complex microbial communities [5].

The development and optimization of next-generation sequencing-based platforms in the past decade have revolutionized the field of genomics, providing several sequencing alternatives that are faster, and more reliable and applicable [7] for ribosomal DNA-based molecular markers, which have many advantages in massive and metagenomic sequencing studies and are considered the basis for the current systematic classification of bacteria and archaea [8]. In addition, the comparison of these sequences represents a powerful tool for deducing phylogenetic and evolutionary relationships between these microorganisms [9,10,11,12]. These culture-independent approaches have been a key component in describing microbiota and microbial food diversity (especially of the most complex fermented foods), providing a great impact on food microbiology [6]. In artisanal dairy products, deep knowledge of the native microbiota not only contributes to their description and understanding but could also serve to select microorganisms and establish the starter-culture dose to improve the transformation steps, increasing the sensory properties of the product [13,14,15,16,17].

Core microbiota of several raw-milk cheeses around the world have been described, such as Gouda cheese (strongly represented by the genera *Streptococcus*, *Lactobacillus*, and *Lactococcus*) [18]; Plaisentif cheese, an Italian handmade semihard cheese (microbiota mainly composed of the genera *Lactococcus*, *Lactobacillus*, and *Streptococcus*) [19]; and Kazak cheese, an artisanal cheese produced through a process called multispecies fermentation (microbial population represented by *Lactobacillus*, *Streptococcus*, *Kluyveromyces*, and *Torulaspora*) [20].

Some Mexican artisanal cheeses [21,22] have been also studied to describe their microbiota through the use of culture-independent strategies. For example, by pyrosequencing on a 454-FLX Titanium platform (Roche Diagnostics Ltd., West Sussex, UK), the bacterial communities of two Mexican cheeses were described: Bola cheese (Ocosingo, Chiapas, México) and Poro cheese (Balancán, Tabasco, México), which are cheeses ripened by the native microbiota of bovine milk [23,24]. The microbiota of the Bola de Ocosingo cheese (protected by a collective trademark) turned out to be strongly represented by *Streptococcus thermophilus*, *Lactococcus lactis*, *Lactobacillus helveticus*, *L. delbrueckii*, and *L. plantarum*, although undesirable microorganisms such as *Escherichia coli* and *Shigella flexnerii* were also found in a low proportion throughout the elaboration process, even though its recovery was not possible by culture-dependent microbiology techniques [25]. Regarding Poro cheese microbiota, a demineralized cheese shredded during its preparation (which causes pore formation inside the cheese) [23,24], it was strongly represented by bacteria of the *Streptococcus* and *Lactobacillus* genera, although *Staphylococcus*, *Acinetobacter*, *Chryseobacterium*, *Bacillus*, *Sediminibacter*, *Lactococcus*, and *Enterococcus* bacteria were occasionally present. After curdling, the dominant microorganisms were *Streptococcus thermophilus* and *Lactobacillus delbrueckii* [14].

Specifically, Adobera cheese from Los Altos de Jalisco is an unripened fresh cheese mainly produced in the Los Altos region of the state of Jalisco in Mexico, one of the most important dairy basins in the country. Adobera has been described as an artisanal raw-milk cheese—acidified by the native microbiota of the cow milk used for its manufacture—that exhibits a low pH and melts upon heating [22,26]. The Adobera cheese-making protocol begins with the milk reception (occasionally standardized to 3% of fat content), followed by enzymatic coagulation (rennet ratio: 1:10,000 milk), curd cut, agitation, and whey removal. One of the essential and distinctive steps of the Adobera cheese-making process is the mass acidification (cheddarization) of the curd over 18 to 24 h at room temperature, causing a significant decrease in the pH (pH values around 5.0), followed by grinding, salting (1–2% NaCl), molding and pressing, resulting in a yellowish-to-ivory semihard cheese with good sensory acceptability-related scores [22,26].

A first report aiming to describe the Adobera cheese microbiota was published by Murugesan et al. [27], pointing out that its microbiota is strongly represented by *Streptococcus*, *Lactococcus*, and *Lactobacillus* bacteria, and some fungi such as *Saccharomyces* sp., *Scheffersomyces* sp., *Galactomyces* sp. However, the study did not include results related to the bacterial-community composition of the milk used in cheese manufacturing or of intermediate stages of the process, which would elucidate the population changes of the bacterial community of this cheese induced by the processing conditions and even factors such as seasonality.

This study aimed to examine the bacterial composition of the artisanal Adobera cheese from Los Altos de Jalisco and the metabolic predicted profile in different stages of the cheese-making process and two seasons of the year.

## 2. Materials and Methods

### 2.1. Samples Collection

Three cheese factories that preserve the original elaboration protocol [22,26] of artisanal Adobera cheese (food-additive free) located in two municipalities from Los Altos de Jalisco region were selected. A total of 26 samples were collected, corresponding to four stages of cheese making: raw milk (RM), fresh curd (FC), acidified curd (MC), and cheese (CH) in both dry (November to May) and rainy seasons (June to October). Samples were stored at 4–7 °C, shipped to the Microbial Genetic Resources Laboratory of the CNRG-INIFAP and frozen at −20 °C until processing. Additionally, two cheese samples from dry-season cheeses were taken and stored (4–7 °C) for 21 days to evaluate changes in the bacterial community through shelf-life.

### 2.2. DNA Extraction

Metagenomic DNA from 0.2 g of each of the collected samples was obtained using a commercial extraction kit (Fecal DNA extraction Kit, Bio Basic Inc., Markham, ON, Canada). The integrity of the genetic material was verified through a 1% agarose gel electrophoresis for 50 min at 80 V. DNA was stored at −20 °C until use in sequencing procedures.

### 2.3. Construction of the 16S rRNA Libraries and High Throughput DNA Sequencing

PCR-based amplification of 7 of the 9 hypervariable regions of the 16S rRNA gene (V2, V3, V4, V6-7, V8, and V9) was performed in two independent reactions using the 16S metagenomics system according to the manufacturer’s instructions (Thermo Fisher Scientific, Waltham, MA, USA) in a SelectCycler device (Select BioProduct, Life Science Research, Waltham, MA, USA). Subsequently, 50 nanograms of an equimolar mixture prepared from the amplification products were used to generate the 16S rRNA libraries by using the Ion Plus Fragment commercial system and Ion Xpress barcode adapters (Thermo Fisher Scientific). Purification was performed in each of the steps with the Agentcourt AMPure XP system according to the manufacturer’s instructions (Beckman Coulter, Brea, CA, USA). Libraries were quantified using the highly sensitive DNA commercial system and Bioanalyzer 2100 (Agilent Technologies, Santa Clara, CA, USA) and the concentration was adjusted to 26 pM. PCR-amplification of the emulsion (PCR emulsion) was carried out in a 25 µL volume from an equimolar mixture of all samples (One-Touch 2, Thermo Fisher Scientific) and enriched in the OneTouch Enrichment system (Thermo Fisher Scientific). Sequencing was performed on the PGM-Ion Torrent (Personal Genome Machine) platform.

### 2.4. Bioinformatics

Sequences data were analyzed using the QIIME (Quantitative Insights Into Microbial Ecology pipeline, v1.9.0) available in Phyton 2.7 for the Ubuntu 17.10 operating system as previously described [28]. The BAM files were first converted into FASTQ files. Sequences were quality-assessed (FastQC, v 0.11.7), trimmed to remove low-quality regions (Trimmomatic 0.36) [29,30], and assigned to operational taxonomic units (OTU). Unassigned sequences were allocated to a de novo algorithm for their grouping (97% correspondence) [31], and phylogenetically representative OTUs were assigned (80% similarity, RPD classifier) [32]. Alpha diversity indexes (observed species, Chao1, Shannon, and Simpson) were calculated from the rarefaction curves. Meanwhile, Beta diversity indexes were calculated through the UniFrac metric system (weighted and unweighted) and visualized through principal coordinate analysis (PCoA) [33] and multivariate analysis, using a linear model to elucidate associations among the microbiota composition and some variables such as seasonality, composition, and stage of the cheese-making process. The derived 16S rRNA gene sequences are available at the NCBI under Bioproject accession PRJNA681198. Finally, predictive functional genomic analysis of the bacterial communities related to Adobera cheese in different process stages and seasons was carried out by PICRUSt [11], based in the Greengene 16S rRNA gene dataset.

## 3. Results

### 3.1. Bioinformatic Analysis

Preparation of DNA libraries was based on simultaneous PCR amplifications of two reactions using the six primers included in the ION 16S Metagenomics kit targeted for the V2, V4, and V8 regions in one reaction, and V3, V6–V7 and V9 regions in the second one. Amplicons were obtained from the 26 samples corresponding to three different cheese factories (Q1–Q3), four different stages of cheese manufacturing (RM = raw milk, FC = fresh curd without acidification, MC = matured curd, and CH = cheese) and two different seasons, dry season (T1) and rainy season (T2). Libraries were sequenced and analyzed as described above.

A total of 4,231,053 readings were obtained, with an average of 162,732 readings per sample (Table 1). After filtering the sequences, 1,196 OTUs (operational taxonomic units, represented by two or more readings) were identified and assigned to 15 phyla, 30 classes, 72 orders, 180 families, and about 400 bacterial genera. Rarefaction curves for the observed species reached a saturation point indicating that the probability of finding new or different OTUs from those found was practically null. Diversity indexes (Chao1, Shannon, Simpson, and Simpson reciprocal) [34] remained constant among the process (Table 1) and only exhibited minor changes, particularly in cheeses from factories 1 and 2; also, differences between seasons were found.

### 3.2. Differences in Microbiota Composition: From Milk to Cheese

Four predominant phyla were found in all samples, corresponding to Firmicutes, Proteobacteria and, in a lower proportion, Actinobacteria and Bacteroidetes. In raw milk, average relative abundance was practically equal for Firmicutes and Proteobacteria (47.22 vs. 47.98%, including dry and rainy season samples). Nonetheless, these proportions changed significantly because Firmicutes reached an average relative abundance of 66.9% in cheese, inducing a decrease in Proteobacteria (32.12%). Firmicutes and Proteobacteria were mostly represented by the Bacilli and Gammaproteobacteria classes (47.08% and 47.20% in raw milk vs. 66.83% and 30.88% in cheese), and by the Lactobacillales, Enterobacteriales, Aeromonadales and Pseudomonadales orders (45.80%, 17.27%, 14.82% and 14.77% in raw milk and 66.55%, 22.10%, 6.33% and 2.18% in cheese). The most representative bacterial families were *Streptococcaceae*, *Enterobacteriaceae*, *Aeromonadaceae* and *Lactobacillaceae* (40.9%, 17.27%, 14.82%, and 0.73% in raw milk vs. 57.60%, 22.10%, 6.33%, and 5.35% in cheese), which were strongly represented by the genera *Streptococcus* spp., *Lactococcus* spp., and *Lactobacillus* spp., as well as unassigned *Enterobacteriaceae* and *Aeromonadaceae* (23.12%, 17.53%, 0.72%, 13.10% and 9.83% in raw milk, and 38%, 19.35%, 5.27%, 17.50% and 4.50% in cheese).

### 3.3. Bacterial Communities of Adobera Cheese during Process

Taxonomic assignation indicated that the most abundant phyla in the majority of the samples were Firmicutes (59.0%, expressed as the average of the total of the samples) and Proteobacteria (38.9%), followed by Actinobacteria (1.2%) and Bacteroidetes (0.8%). Changes in bacterial populations associated with different stages of the cheese-making process showed that the relative abundance of Proteobacteria decreased while Firmicutes increased. In some cases, Proteobacteria registered a higher relative abundance in contrast to Firmicutes in samples corresponding to the early stages of the cheese-making process (raw milk and fresh curd), but at the end, Firmicutes was always the predominant phylum, representing up to 78% of the bacterial community. This trend continued during the refrigerated storage of cheese, as noted in the 21-day samples, in which Firmicutes reached up to 83% in terms of relative abundance, while Proteobacteria decreased to 20% of relative abundance.

Bacterial classes were represented by Bacilli (58.9% average of the total samples), followed by Gammaproteobacteria (38.3%), especially in samples corresponding to the final stages of the cheese-making process (matured curd and cheese). The Actinobacteria, Flavobacteria, Alphaproteobacteria, and Betaproteobacteria classes were less than 1% and, as we observed at the phylum level, bacterial populations also registered changes during cheese shelf-life and in the 21-day samples under refrigerated storage, they were represented only by Bacilli (82%) and Gammaproteobacteria (16%).

The most representative groups of bacteria, at order level, were those belonging to the Lactobacillales order (58.2% relative abundance in the total assigned sequences), followed by the Enterobacteriales (20.4%), Aeromonadales (10.5%), and Pseudomonadales (7.1%). Curd acidification favored an increase of Lactobacillales, reaching a relative abundance of 69.9% in the dry-season cheese and 63.2% in its rainy counterpart. In turn, bacteria from the Enterobacteriales, Aeromonadales, and Pseudomonadales orders decreased to 17.4%, 7.6%, and 3.4%, respectively, in the dry-season cheese samples, and 26.8%, 5.1%, and 1%, respectively, for cheese in the rainy season, regarding what was found in raw milk of the same samples. Similarly, cold storage modified the relative abundance in the cheese bacterial community, increasing Lactobacillales (82.9%) in dry-season samples after 21 days post-processing, and reducing Enterobacteriales (14.9%), Aeromonadales (0.8%), and Pseudomonales (0.5%). The families of bacteria that had the greatest abundance in Adobera cheese samples were *Streptococcaceae* (50.5% average in all samples), *Enterobacteriaceae* (20.4% average), *Aeromonadaceae* (10.5%), *Moraxellaceae* (4.1%), *Lactobacillaceae* (3.7%), and *Pseudomonadaceae* (3.0%).

As occurred with the first taxonomic levels, the cheese-making stage and the season in which cheese was produced (seasonal effect) affected the relative abundance within families. The dominant family in milk was *Streptococcaceae* (47.5% in the dry season vs. 34.3% in the rainy season), which had a higher abundance after curd acidification (62.8% vs. 52.4% for cheese in the dry and rainy seasons). Cold storage also modified the relative abundance in cheese at the family level and, after 21 d postmanufacture, *Streptococaceae* reached values around 70% of relative abundance in dry-season cheese samples. The presence of the *Lactobacillaceae* family also increased after curd acidification, reaching relative abundance of 4.1% and 6.6% in cheese for dry and rainy seasons, respectively. A slight increase was also observed for the *Enterobacteriaceae* family (17.4% and 26.8% for cheese in the dry and rainy seasons), while the rest of the families decreased due to the manufacturing process.

**Figure 1:**
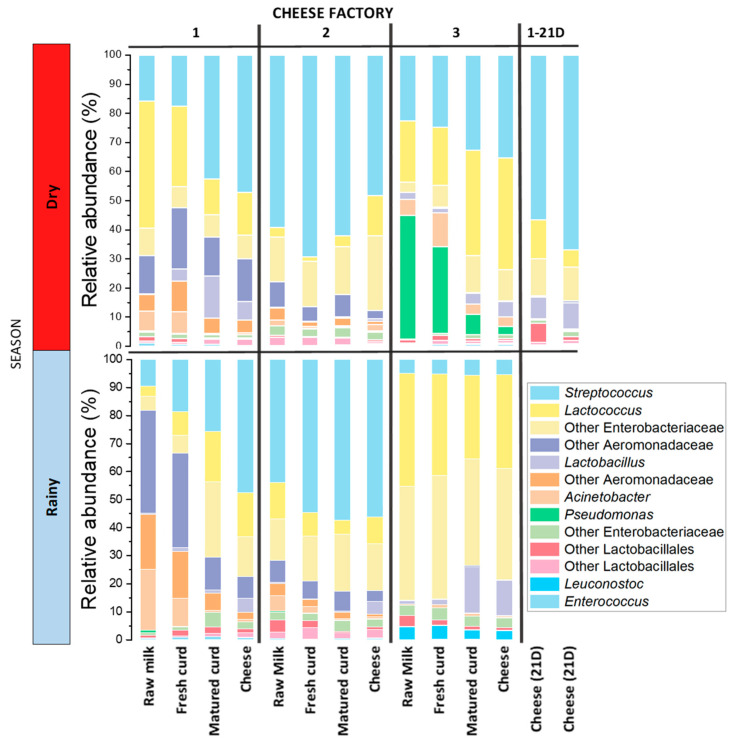
Relative abundance of main bacteria genera conforming artisanal Adobera cheese microbiota samples at different stages of processing and different sampling seasons. 1–21: Cheese samples from dry season preserved 21 days under refrigeration.

At genus level, the genera found in a greater relative abundance were *Streptococcus*, *Lactococcus*, *Lactobacillus*, and *Pseudomonas* (Figure 1). Population succession was similar to that observed at other taxonomic levels. Lactic-acid bacteria belonging to Firmicutes (*Streptococcus*, *Lactococcus*, *Lactobacillus*, *Enterococcus,* and *Lactococcus*) increased after curd acidification and remained as the main genera in cheese at the end of the process (41.4%, 21.1%, 4%, and 0.43%, respectively, for dry-season cheese samples, and 34.6%, 17.60%, 6.53%, and 0.4%, respectively, for rainy-season cheese samples), representing over 70% of the bacterial community in the cheese. Regarding Proteobacteria, the prevalent genera of this phylum in cheese were *Acinetobacter* (1.9% and 0.63% for dry and rainy seasons), *Pseudomonas* (1.03% and 0.10% for dry and rainy seasons), *Serratia* (0.43% and 2.13% for dry and rainy seasons), and *Citrobacter* (0.50% and 0.43% for dry and rainy seasons).

### 3.4. Factors Driving Changes in the Bacterial Community of Artisanal Adobera Cheese

**Figure 2:**
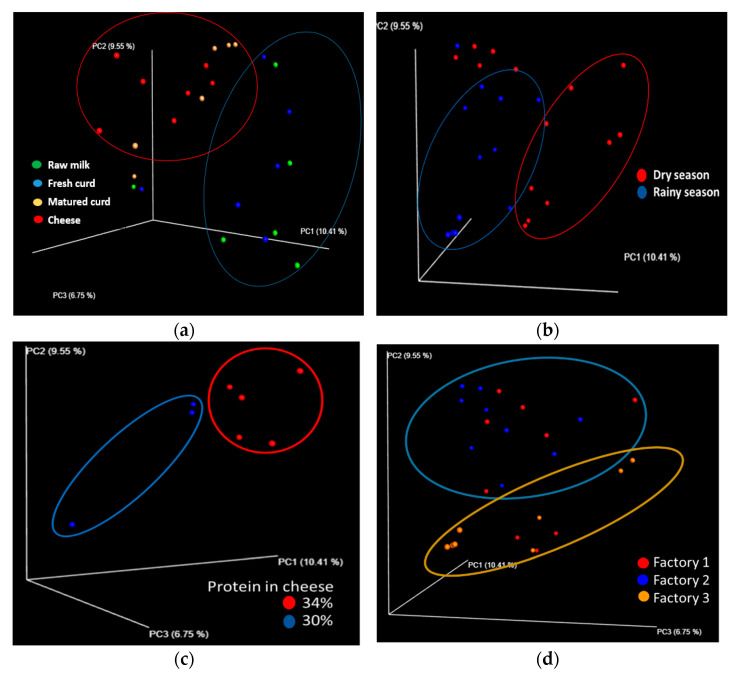
Beta diversity for artisanal Adobera cheese samples shown in a principal coordinates chart from: (**a**) different stages of preparation and two seasons. The letter Q represents the cheese factory number; letter T the season of the year (T1 = dry season, T2 = rainy season). Raw milk = raw milk; Fresh curd = fresh curd; Matured curd = ripened or acidified curd; Cheese = cheese as a finished product (**b**) Dry and rainy seasons (**c**) different protein content in cheese, and (**d**) three different cheese factories.

**Figure 3:**
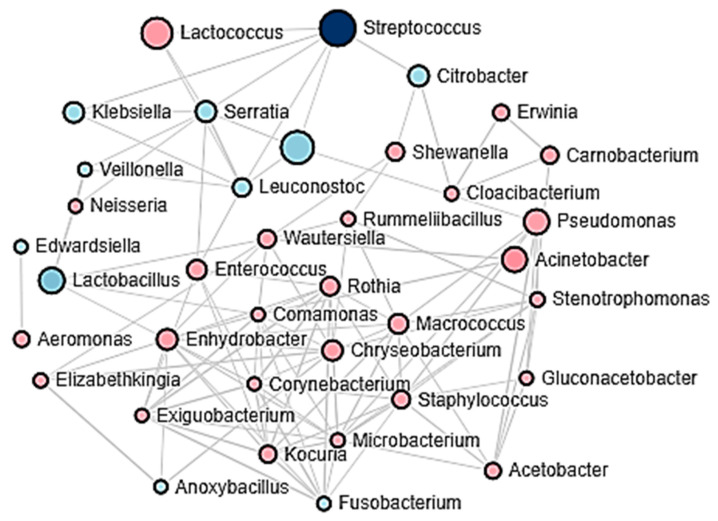
Schematic representation of the interaction among the bacterial genera with the highest abundance in raw milk vs. their persistence in artisanal Adobera cheese based on Pearson’s correlation analysis (*p* < 0.05, *p* > 0.3). Each circle represents a different bacterial genus. Larger circles represent correlations of greater magnitude and a greater number of correlations with other bacterial genera. The lines connect each bacterial genus with those it correlates with.

Changes associated with the stage of cheese elaboration process and seasonal effects on the structure of the bacterial communities in Adobera cheese samples, evaluated through a principal component analysis (PCoA), are shown in Figure 2a,b, as well as differences associated with protein content in cheese (Figure 2c) and the cheese factory (Figure 2d). On the other hand, Pearson’s correlations, among main bacterial genera in Adobera cheese’s core microbiota, are schematized in Figure 3.

**Figure 4:**
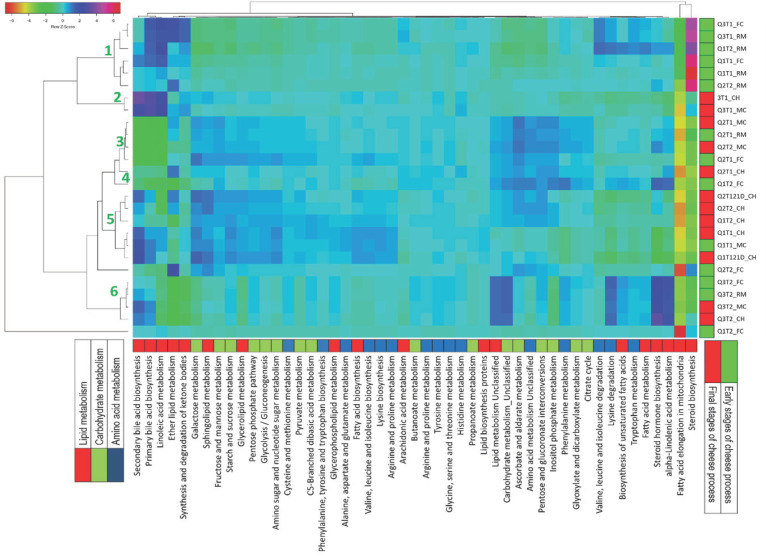
Heap plot representation of the predicted metabolic profiles related to carbohydrate, amino acid, and lipid metabolism in Adobera cheese samples from different cheese factories (CH1, CH2 and CH3) and two seasons of the year (T1 = Dry season, T2 = Rainy season) at different stages of process (RM = raw milk, FC = fresh curd, MC = matured curd, CH = cheese). CH_21D = cheese 21D after elaboration. Rows and columns were clustered by the centroid linkage method and distances were measured by Pearson’s correlations and represented by colors according to the degree of correlation among samples and the predicted relative frequency of genes.

Regarding predictive metabolism, 327 KEGG pathways were predicted through the inclusion of all samples. At level two of the functional subcategory, the predicted genes were related to four principal types of pathways, namely membrane transport, amino acid metabolism, lipid metabolism, and carbohydrate metabolism. Predicted pathways were filtered to obtain and analyze predicted metabolic profiles related to carbohydrates, lipids, and amino acid metabolism associated with the microbiota composition in the different cheese samples (Figure 4).

**Figure 5:**
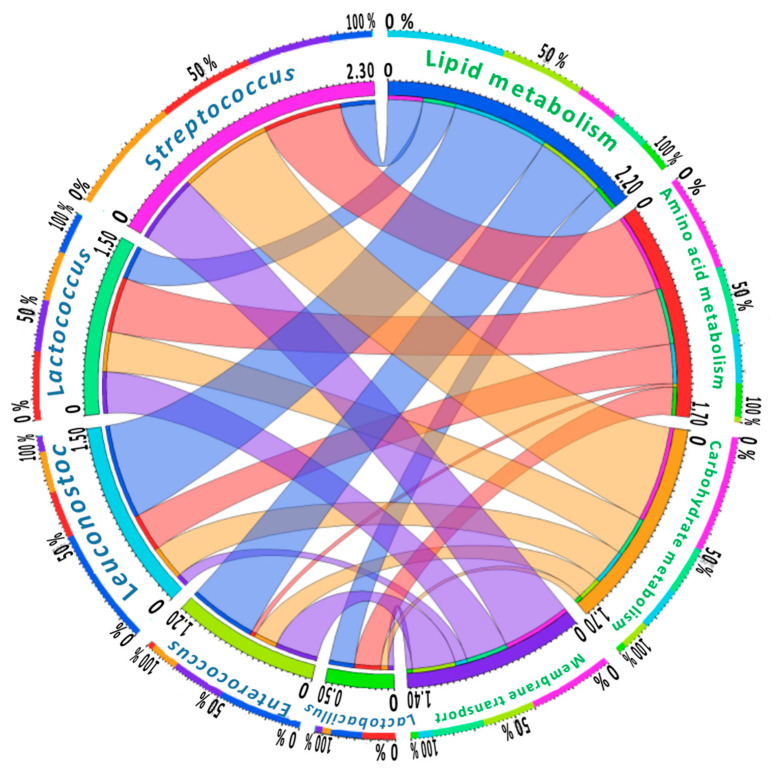
Schematic representation of Pearson’s correlations among relative abundance of main genera of core bacteria and relative frequencies of KEGG predicted genes associated with metabolic pathways at level two in artisanal Adobera cheese samples. Stronger correlation scores are represented by thicker bonds (Created with: Circos Table Viewer v0.63-9).

Finally, correlation analysis showed that the relative abundance of the main lactic-acid bacteria-related genera present in core Adobera cheese microbiota correlated with the relative frequency of the main metabolic predicted pathways. For example, *Streptococcus* positive correlated (*p* < 0.05) with membrane transport pathways, amino acid and carbohydrate metabolism, while the lipid metabolism seemed to be mainly driven by *Leuconostoc* (Figure 5).

## 4. Discussion

Amplicon-based profiling has been successfully used to follow the succession of microbial populations over time at various taxonomic levels and is a popular tool for culture-independent studies of food-related microbial communities, including several types of cheese [35,36]. Data obtained from the microbial communities represent a powerful tool with great potential to determine food safety, by simultaneously detecting pathogenic microorganisms (including retrospective studies of outbreaks associated with food), establishing the biotechnological potential of the microbial communities, serving as the basis for process standardization involved in the production of artisanal food products, and identifying batch and seasonality effects on the quality of artisanal dairy products, among others [36,37,38,39,40].

Bacterial communities involved in the production of only some genuine Mexican cheeses have been described [14,25,27,41]; however, reports about the microbiota of artisanal Adobera cheese [26] are very scarce, so there are no studies of its succession during the process or about the effects of seasonality that could determine cheese composition and characteristics. In this study, we used a high-throughput approach to describe bacterial communities taking part in Adobera cheese production, strongly represented by Firmicutes- and Proteobacteria-related bacteria, mainly lactic-acid bacteria-associated genera.

Murugesan et al. [27] reported that the bacterial community of the Adobera melting cheese was composed of the *Streptococcus*, *Lactococcus*, *Lactobacillus*, *Clostridium,* and *Aeromonas* genera, which coincided with our results. The differences observed between raw milk and cheese were also similar to that reported for *Poro* and *Bola de Ocosingo* cheeses, two artisanal and ripened Mexican cheeses [14,25]. Modifications postmanufacture under cold storage also induced changes in relative abundance at the genus level, favoring the increase of lactic-acid bacteria-related genera. 

The bacterial community of artisanal Adobera cheese observed in this study is similar to other bacterial communities, especially of hard and semihard cheeses, including some Mexican varieties [15,25,27,42] and even some ripened cheeses such as Plaisentif, an Italian raw-milk cheese [19]. Core bacterial community members of Adobera cheese (final product) comprise *Streptococcus*, *Lactococcus* and *Lactobacillus*; additionally, other unclassified *Lactobacillales* (not family- or genus-assigned) were detected in cheese samples. However, as we observed, lactic-acid bacteria-related genera were present since the beginning in the raw milk and through the entire manufacturing process. In this context, it has been reported that raw cow’s milk microbiota includes several species of microorganisms, mainly related to the animal, especially on the udder surface. Nonetheless, fresh milk microbiota is also enriched by cross-contamination from other sources, such as those related to environmental conditions under which the milk is produced and surfaces involved in milk manipulation, storage, and transport (milking machines, storage tanks, operators’ hands, dust, forages, etc.) [43,44]. All these combinations of factors define milk’s microbiota, which have been reported as heterogeneous but mainly integrated by lactic-acid bacteria, including *Lactobacillus* spp., *Lactococcus* spp., *Streptococcus* spp., *Enterococcus* spp., and *Leuconostoc* spp., which, individually, can be in viable counts from 10^1^ to 10^4^ CFU/mL [5,44,45]. 

Milk microbiota are crucial in defining artisanal-cheese microbiota since there will be no pre-processing treatment that will reduce the bacterial content (e.g., pasteurization); especially, the presence of lactic-acid bacteria is fundamental for driving the fermentation process required in cheese manufacturing [15]. Particularly, lactobacilli, which could include several hetero- and facultative hetero-fermentative strains [46], could contribute to cheese acidification in the early stages of the process and to flavor development in the final stages and storage [47]; actually, some lactobacilli strains are used as adjunct cultures. Some Streptococci strains (not commonly used in food fermentations) in cheese have been recognized as starter cultures (native or added) to produce hard and semihard cheeses, and are considered as persistent bacteria in artisanal cheeses [47,48]; however, only *S. thermophilus* strains have been used as starter cultures and occur naturally in some artisanal cheeses as well as some *Streptococcus gallolyticus* strains [49]. Finally, *Lactococcus*, the third member of the core microbiota, has extensively been used to produce dairy products. Lactococci strains work as a starter lactic-acid bacterium, inducing cheese acidification, but they also can play a key role in protein hydrolysis, contributing to cheese texture and flavor development, producing organic acids [50].

Secondary members of the Adobera cheese core microbiota included *Leuconostoc* and *Enterococcus*. These genera act as nonstarter lactic-acid bacteria and could explain why we observed an increase in these genera at the final steps of the Adobera cheese manufacturing. *Leuconostoc* strains have a particular role in cheese and fermented dairy product manufacturing, one of their principal activities is related to the development of buttery flavor associated with diacetyl produced by citrate metabolism when the pH reaches low values, although CO_2_, also related to the use of citrate, is another metabolism product of *Leuconostoc* (important in the eye formation in cheeses such as Gouda). Finally, *Leuconostoc* is also important in cheese to remove acetaldehyde excess produced by lactococcal strains used as starter cultures [51]. On the other hand, *Enterococcus* (originally considered part of the *Streptococcus* genus), is important in cheese manufacture due to its ability to hydrolyze proteins and fat, but also for the ability to metabolize citrate. Those metabolic activities directly contribute to aroma and flavor development, although the yield and capabilities are strain-dependent, and some of the enterococci strains also have been catalogued as bacteriocin producers and even as probiotics [52].

Finally, some undesirable bacterial genera remained present (in very low proportions) as core microbiota in Adobera cheese, including *Acinetobacter* and *Pseudomonas* and other nonassigned *Enterobacteriaceae*. *Acinetobacter* spp., have been isolated from several matrices (including foods) and occur naturally in human skin, but have been related to nosocomial infections, mainly caused by *A. baumannii*. However, only *A. johnsonni* and *A. lwoffi* are predominant in food [53], and some other non-*baumannii* strains have been isolated from raw milk and raw-milk cheeses [54,55]. Nonetheless, *Acinetobacter* spp., have been detected in the core and surfaces of matured cheeses and, due to their tolerance to low pH and high salt concentrations, it has been hypothesized that could contribute to the development of sensory characteristics [56]. Moreover, *Pseudomonas* spp. are considered a psychrotolerant bacterial contaminant related to spoilage of milk and dairy products; their incidence in raw-milk cheeses is associated with inadequate procedures of cleaning and sanitation (applied to milking machines, utensils, storage tanks, pipelines, and curding vats among others), via biofilm or by the use of contaminated water [57,58,59]. 

Enterobacteriaceae is usually (but not exclusively) associated with fecal contamination of raw milk, but also by cross-contamination during milk manipulation and processing. This family includes several bacterial genera considered as potential pathogens (such as *Salmonella* and *Escherichia*) and could induce early spoilage and potential food-unsafe outbreaks; however, several factors such as pH, water activity, or salt content can reduce the survival of this group of bacteria, commonly used as hygienic quality indicators [60]. Specifically, for artisanal Adobera cheese, it has been hypothesized that pH could serve as a control strategy of this type of microorganism because of the low incidence of pathogen strains in cheeses with pH values below 5.0 [61].

However, as our results showed, the major number of reads assigned to undesirable bacterial genera were found in the early stages of Adobera manufacturing (raw milk and fresh curd), but their relative abundance was lower in cheese as a final product, possibly associated with the selection pressure that the manufacturing process and the rest of the microbiota members wield during cheese production. Despite this, we do not necessarily imply that these microorganisms are viable or metabolically active.

A peculiarity of Adobera cheese is that despite being a cheese with a high moisture content, it melts upon heating due to the cheddaring involved during its manufacturing [26]. This acidification process (that takes up to 24 h) seemed to be a crucial step to define the cheese core microbiota, favoring the proliferation of lactic-acid bacteria and limiting the survival of undesirable groups of bacteria. This was corroborated through the microbiota beta diversity at different cheese-manufacturing steps (Figure 2a). On the other hand, there seemed to be a seasonal effect on the structure of the bacterial communities in Adobera cheese samples (Figure 2b). This could be intimately related to the microbiological quality of the milk used for the process, as well as the conditions under which it was obtained. Otherwise, changes have been reported between seasons on the milk composition (fat, protein, casein, and lactose) [62]; these changes are mainly associated with fresh-forage availability in the rainy season that directly affects the technological properties of milk and the efficiency in solids recovery and syneresis and, therefore, cheese composition. Specifically, we observed that cheese-protein content could play a key role on the microbiota structure because cheeses in rainy seasons registered a lower protein content in comparison with their dry-season counterparts (30% and 34%, estimated through FTIR, data not shown). In a previous study, we also observed higher proteolysis in artisanal Adobera cheese sampled during the rainy season [26]. 

We also observed changes in the bacterial community among cheese producers (Figure 2d), which were expected due to the artisanal origin of cheese and slight differences in the process such as curding time or temperature, fat-content standardization, and cheddaring conditions. The structure of the bacterial community observed in cheese samples from factories 1 and 2 was very similar, while cheese from factory 3 showed a lower abundance of streptococci and a higher proportion of nonassigned Enterobacteriaceae, possibly associated with poor hygiene practices or a shorter acidification time during cheese manufacture. 

Finally, the Pearson’s correlation analysis showed that certain bacterial genera had positive and negative correlations with other genera, which favor or limit the incidence of certain bacterial groups. The analysis showed that the genera that had the greatest interaction and influence in defining the structure of the bacterial community in Adobera cheese were *Streptococcus*, *Lactococcus*, *Lactobacillus*, *Pseudomonas*, and *Acinetobacter* (Figure 3). Lactic-acid group-related bacteria strongly contributed to organic-acid production, inducing a significant pH decrease and limiting the survival of undesirable bacteria, hence the importance of guaranteeing an adequate proportion of these bacteria in raw milk at the beginning of cheese production. 

The predicted metabolism profile showed that, at level two of the KEGG functional subcategory, the predicted genes were related to four principal pathways, namely membrane transport, amino-acid metabolism, lipid metabolism, and carbohydrate metabolism. Regarding carbohydrate, lipid and amino-acid metabolism associated with the microbiota composition in the different cheese samples (Figure 4), at least six clusters were distinguished, and major lipid-metabolism-related genes were observed in the early stages of cheese manufacture such as linoleic acid and ether-lipid metabolism, which increased at the final stages of process elaboration, along with others such as secondary bile-acid biosynthesis and some carbohydrate (i.e., galactose and fructose) and amino-acid metabolism and catabolism pathways. However, from the total of predicted genes, only 28 showed statistical differences among samples (*p* < 0.05), three associated with carbohydrate metabolism (fructose and mannose metabolism, pentose and glucoronate interconversions), two related to amino-acid metabolism (tyrosine and other amino-acid metabolism) and two more to lipid metabolism (glycerolipid metabolism and primary bile-acid biosynthesis). The remaining significant metabolic pathways were mainly related to transporters, phosphotransferase system (PTS), carbon fixation in photosynthetic organisms, sulfur metabolism, biosynthesis of ansamycins, betalain biosynthesis and RNA degradation, among others. It was interesting to find predicted genes involved in xenobiotics biodegradation and metabolism (specifically to DDT degradation), mainly in raw-milk samples. 

It has been reported that *Streptococcus* strains may contribute to galactose fermentation, the production of essential amino acids, and exopolysaccharides, attractive attributes from a technological point of view [63]; for this reason, some members of this genus (particularly *Streptococcus thermophilus* strains) have been proposed to be used as starter cultures in cheese elaboration. Tidona et al. [64] reported that a combination of *Streptococcus thermophilus* strains, selected mainly for their metabolic characteristics as phage resistance, acidifying capacity, growth temperature, and NaCl tolerance, exhibited a good performance when they were incorporated as starter cultures in Crescenza cheese manufacturing, expressed in terms of technological behavior and cheese quality. Although *Streptococcus* is not recognized as a proteolytic genus, some proteolytic strains were included (based on their capability to hydrolyze urea). This supports the idea that, in Adobera cheese, *Streptococcus* not only may contribute to carbohydrate metabolism but also to protein and amino-acid hydrolysis and use. 

*Lactococcus* has been reported as one of the predominant genera in some cheeses, including Oaxaca cheese, and its metabolic contributions to cheese production are important to elucidate. Specifically, *Lactococcus lactis* strains isolated from Oaxaca cheese have been screened, showing high intraspecies diversity (using molecular-fingerprints approaches), and some of these strains exhibited important technological properties such as antibiotic resistance, good acidifying capability, and the ability to produce volatile compounds such as 3-methylbutanal—characteristics that could be exploited to use those strains as starter cultures for cheese production [65]. However, the role of the autochthonous lactic-acid-bacteria metabolism involved in cheese production is still under study, mainly associated with the versatility of these bacteria from a metabolic point of view and the evidence of their active contribution not only to primary metabolism, but their contributions for the accumulation of some compounds such as short-chain fatty acids that contribute to flavor development and benefit the consumer by inducing protection against some types of cancer and other disorders and diseases [66,67,68].

Some perspectives can be pointed out according to our results, mainly related to the fact that raw-milk cheeses are distinguished from pasteurized-milk cheeses by a strong, intense, and distinctive flavor, largely associated with autochthonous bacteria involved in their fermentation and maturation (aged cheeses) and, in some cases, these bacteria also ensure the safety of the cheeses by eliminating the main pathogens [69,70]. Otherwise, autochthonous lactic-acid bacteria isolated from raw-milk cheeses have been exploited as starters or adjunct cultures to improve or standardize their elaboration processes [71,72], and their ability to inhibit other microorganisms has allowed them to eliminate pathogens from raw-milk cheeses such as *Salmonella*, *Staphylococcus aureus*, and *Listeria monocytogenes* [73,74,75], or even to decontaminate more complex matrices such as swine manure [76]. Regarding the probiotic potential that could be explored in Adobera cheese, several strains of lactic-acid bacteria isolated from raw-milk cheeses (mainly *Lactobacillus*, *Lactococcus,* and *Enterococcus* strains) have been used as probiotic bacteria, including some autochthonous bacteria from Mexican cheeses [77,78,79,80].

## 5. Conclusions

Next-generation sequencing allowed the identification of the core bacterial community of Adobera cheese, mainly dominated by *Streptococcus* spp., *Lactococcus* spp., and *Lactobacillus* spp. This is the first study describing seasonal effects and bacterial succession in the microbiota of artisanal Adobera cheese through its elaboration process, providing evidence that acidification has a key role in defining the cheese core microbiota by limiting the proliferation of undesirable bacterial genera. Meanwhile, the metabolic predicted profile was dominated by amino acid, carbohydrate, and lipid metabolisms as well as membrane-transport-related pathways. Our results could be used as a basis to design strategies to standardize and improve the safety of artisanal Adobera cheese. Furthermore, the bacterial community involved in the manufacturing of this genuine Mexican cheese could be explored through other approaches, including the isolation and characterization of lactic-acid bacteria, to establish their technological and probiotic potential, and their inclusion in biotechnological developments.

## Figures and Tables

**Table 1:** Number of sequences and diversity estimators for Adobera cheese samples.

Cheese	Season	Sample	Valid Reads	Chao1	Shannon	Simpson	Simpson Reciprocal
1	Dry	Raw Milk	112,441	3954.71	6.539	0.948	19.23
		Fresh curd	41,138	4143.00	6.821	0.968	31.09
		Matured curd	299,377	4304.96	6.128	0.967	29.95
		Cheese	480,795	4525.90	6.105	0.965	28.78
	Rainy	Raw Milk	69,215	3049.56	6.199	0.962	34.76
		Fresh curd	336,678	4719.12	6.250	0.955	22.20
		Matured curd	90,918	5156.78	6.957	0.979	47.70
		Cheese	8674	24.20	3.102	0.878	8.26
2	Dry	Raw Milk	516,917	5170.39	6.687	0.978	45.62
		Fresh curd	293,445	4477.34	6.202	0.965	28.50
		Matured curd	183,288	4293.72	6.417	0.947	38.15
		Cheese	106,058	5357.36	6.906	0.974	38.26
	Rainy	Raw Milk	50,256	3774.49	7.071	0.984	62.72
		Fresh curd	38,019	3350.41	6.833	0.981	51.78
		Matured curd	99,925	4379.79	6.545	0.975	39.50
		Cheese	75,107	4554.86	6.744	0.977	43.23
3	Dry	Raw Milk	119,219	4075.30	6.590	0.971	33.94
		Fresh curd	67,669	4417.30	7.011	0.981	51.32
		Matured curd	98,183	6429.53	7.041	0.972	35.32
		Cheese	61,937	5861.76	6.970	0.971	34.46
	Rainy	Raw Milk	41,368	1741.43	5.351	0.949	19.57
		Fresh curd	58,128	2746.97	5.639	0.950	19.92
		Matured curd	17,047	1252.38	5.873	0.956	22.55
		Cheese	10,540	1196.24	5.919	0.958	23.54
21 d post-elaboration							
1	Dry	Cheese	92,545	4453.98	6.179	0.968	31.25
2	Dry	Cheese	67,213	4559.55	6.636	0.974	38.10
